# Pulse Duration Dependent Asymmetry in Molecular Transmembrane Transport Due to Electroporation in H9c2 Rat Cardiac Myoblast Cells In Vitro

**DOI:** 10.3390/molecules26216571

**Published:** 2021-10-30

**Authors:** Tina Batista Napotnik, Damijan Miklavčič

**Affiliations:** Faculty of Electrical Engineering, University of Ljubljana, Tržaška Cesta 25, 1000 Ljubljana, Slovenia; Damijan.Miklavcic@fe.uni-lj.si

**Keywords:** electroporation, calcium uptake, electric field direction, nanosecond pulses

## Abstract

Electroporation (EP) is one of the successful physical methods for intracellular drug delivery, which temporarily permeabilizes plasma membrane by exposing cells to electric pulses. Orientation of cells in electric field is important for electroporation and, consequently, for transport of molecules through permeabilized plasma membrane. Uptake of molecules after electroporation are the greatest at poles of cells facing electrodes and is often asymmetrical. However, asymmetry reported was inconsistent and inconclusive—in different reports it was either preferentially anodal or cathodal. We investigated the asymmetry of polar uptake of calcium ions after electroporation with electric pulses of different durations, as the orientation of elongated cells affects electroporation to a different extent when using electric pulses of different durations in the range of 100 ns to 100 µs. The results show that with 1, 10, and 100 µs pulses, the uptake of calcium ions is greater at the pole closer to the cathode than at the pole closer to the anode. With shorter 100 ns pulses, the asymmetry is not observed. A different extent of electroporation at different parts of elongated cells, such as muscle or cardiac cells, may have an impact on electroporation-based treatments such as drug delivery, pulse-field ablation, and gene electrotransfection.

## 1. Introduction

Exposure of cells to electric pulses of adequate amplitude and durations leads to transient increase in their plasma membrane permeability and the phenomenon is termed electroporation (EP). Increased plasma membrane permeability allows transmembrane transport of otherwise impermeant molecules into cells [1]. With moderate electric fields, cells recover after electroporation and remain viable (reversible EP), however, with stronger electric fields or longer pulse duration, cells do not recover from the damage and they die (irreversible EP) [2,3]. Reversible and irreversible EP is nowadays used in numerous applications in medicine [4], food technology [5], and biotechnology [1].

Electroporation can also be used for intracellular drug delivery. Since it is a physical method, it bypasses the potential safety issue of viral vectors [4]. As a drug delivery method, electroporation has already been used for the delivery of chemotherapeutics to treat tumors (electrochemotherapy) [6], transdermal drug delivery [7], gene electrotransfer for gene therapy and DNA vaccination [8], and for delivery of CRISPR-Cas9 components for gene editing [9]. Electroporation for drug delivery can be controlled by choosing appropriate electric pulse parameters for specific cells and tissues, as well as molecules to be delivered [10], therefore it is of great importance to thoroughly explore the phenomenon.

The transport of molecules through permeabilized plasma membrane occurs presumably through aqueous pores that form in the process of electroporation [11]. The transport takes place during and after the pulse, the latter being dominant at least in the case of small molecules [12,13,14,15,16,17]. However, molecular mechanisms related to cell membrane permeability and transport post pulse are yet to be elucidated, since the pore closure time in molecular dynamics simulations is several orders of magnitude shorter than experimentally determined membrane resealing times [13].

The influx of ions through a permeabilized plasma membrane can also be an indicator of EP [18]. Monitoring the uptake of calcium ions (Ca^2+^) is a very convenient way of evaluating the extent of EP, because calcium concentration in cells is kept very low (around 100 nM) due to its signaling role [19]. Due to the small size of Ca^2+^ ions compared to other EP detection dyes such as propidium iodide (PI) or YO-PRO-1, this method is considered very sensitive and can detect smaller pores, such as those of nanometer size that emerge in EP with ns pulses, as well as lower levels of membrane electroporation [20,21,22]. However, Ca^2+^ can also be transported through voltage-gated calcium channels (VGCC) or other channels [23,24,25]. Moreover, an increase of internal Ca^2+^ concentration (especially in the case of electroporation with nanosecond electric pulses, nsEP) can be a result of its release from internal stores [26,27,28] and amplification with more complex Ca^2+^ pathways, such as calcium-induced calcium release [29,30] and store-operated (capacitive) calcium entry [31]. Nevertheless, in EP with moderate and longer µs and ms pulses, the majority of Ca^2+^ comes to the cell interior from the extracellular medium through EP pores [12,27,32] and can thus be used for EP detection.

When a spherical cell is exposed to a homogeneous electric field, an induced transmembrane voltage (ITV) which varies with position on the membrane is observed: it reaches its peak values at the poles of the cell facing the two electrodes [33,34]. Consequently, EP and uptake of molecules are greater at poles [33]. Numerous studies have demonstrated that the EP at the poles is asymmetrical. However, different asymmetry in uptake of ions and dye molecules was reported in different studies: the uptake was greater at the negative (cathodal) [26,35,36,37,38,39,40] or positive (anodal) [12,22,39,41,42,43,44,45,46,47,48,49,50,51,52,53] side of the cell. The reasons for different asymmetry in experiments are not yet understood, but were suggested to depend on probing molecule size [40,54] and charge [39,55], poration medium composition [40,43], pore size and opening/closing dynamics at poles [35,40,54], cell type and their asymmetric membrane structures [35], and pulse parameters [22,35,45,56].

Orientation of cells in electric field is important for electroporation and, consequently, for transport of molecules through permeabilized plasma membrane. We have previously shown that elongated cells oriented parallel and perpendicular to electric field are not uniformly electroporated when exposed to electric pulses of different durations [32]. Therefore, we decided to further test the hypothesis that pulse duration affects asymmetry of polar uptake of ions in H9c2 rat cardiac myoblast cells that are of elongated shape. We also tested the hypothesis that by lowering the electric field strength of the pulse (just above the threshold for electroporation) we can reverse the polar asymmetry, since it was suggested that different amplitudes may lead to the different opening of pores on anode (+) and cathode (−) side of cells and thus be responsible for different asymmetric molecular transport [35,54]. A different uptake of ions and molecules at cell poles after exposing the cells to electric pulses of different parameters, e.g., duration, may have an important effect on electroporation-based treatments on elongated cells such as heart and muscle cells.

## 2. Results

### 2.1. Comparing Fast and Slow Image Acquisition Modes to Monitor the Changes in Relative Calcium Concentration in Cells after Pulse Exposure

H9c2 cells were exposed to pulses of different pulse duration: single pulses of 100 ns (40 kV/cm), 1 μs (2500 V/cm), 10 μs (1000 V/cm), and 100 μs (400 V/cm) (for electrode configurations see Supplement, Figure S1). The concentration of calcium ions (Ca^2+^) in cells was monitored with a fluorescent calcium indicator Fura-2. The concentration of Ca^2+^ in intact cells is maintained very low (around 100 nM). After electric pulse exposure, intracellular concentration of Ca^2+^ was increased due to Ca^2+^ uptake from external medium (DMEM, 1.8 mM Ca^2+^) and/or the release of Ca^2+^ from internal stores (endoplasmic reticulum, mitochondria) which resulted in Fura-2 response (increased Fura ratio 340/380). To obtain a comparable intracellular Ca^2+^ concentration, as indicated by Fura-2 response, a much higher electric field had to be applied when cells were exposed to shorter pulses than when longer pulses were used [21,32]. 

**Figure 1:**
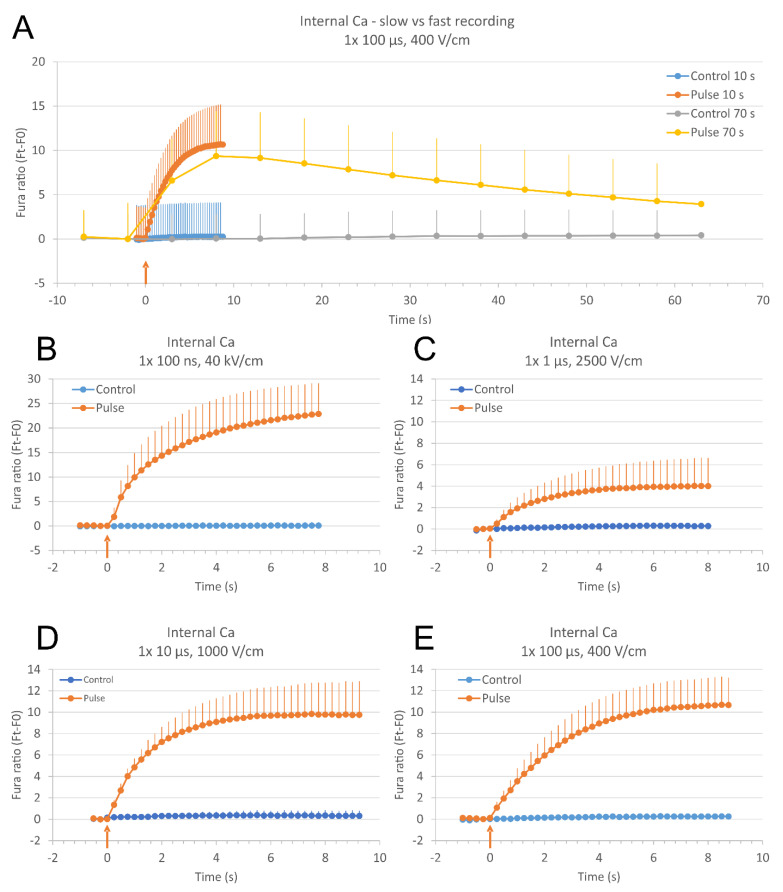
Relative calcium ion (Ca^2+^) concentration in cells after electroporation with single pulses of different duration as monitored with a fluorescent calcium indicator Fura-2 and expressed in Fura-2 ratio 340/380 over time (subtracted baseline). A higher Ca^2+^ concentration results in a higher Fura-2 ratio 340/380. (**A**): Comparing fast- and slow-acquisition modes of calcium ions (Ca^2+^) detection in electroporated cells exposed to a single 100 μs, 400 V/cm pulse. Fast-acquisition mode (10 s, solid lines): total duration of image acquisition: 10 s, time interval between images: 250 ms, number of recorded images: 41. Slow-acquisition mode (70 s, dashed lines): total duration of image acquisition: 70 s, time interval between images: 5 s, number of recorded images: 15. Slow-acquisition mode was done on the same cells 15 min after fast-acquisition, allowing the cells to reseal. Time of pulse application is adjusted to the same pulse delivery time (noted with a red arrow). Results are presented as mean from four experiments (15–19 cells per experiment analyzed). (**B**–**E**): Relative Ca^2+^ concentration in cells after electroporation with a single 100 ns, 40 kV/cm (**B**), 1 μs, 2500 V/cm (**C**), 10 μs, 1000 V/cm (**D**), and 100 μs, 400 V/cm pulse (**E**) as expressed in Fura-2 ratio 340/380 over time. Image acquisition was done every 250 ms. Results are presented as mean from nine (**B**), six (**C**), five (**D**), and four (**E**) experiments (5–27 cells per experiment analyzed, all cells were taken into account). Vertical bars represent SD. Controls are experiments without pulse application.

Relative Ca^2+^ concentration in cells was monitored in a fast-acquisition mode to see the fast events immediately after the pulse application: total duration of image acquisition: 10 s, time interval between images: 250 ms, number of recorded images: 41. Since the complete resealing and low Ca^2+^ concentration restoration (pumping of Ca^2+^ out of the cell and into the internal stores) takes several minutes [57], we compared results from the fast-acquisition mode to those from a slow-acquisition mode (images were taken every 5 s during 70 s total duration of image acquisition) that we used in our previous study [32]. We performed these experiments using 100 μs, 400 V/cm pulses (Figure 1A) where fast and slow image acquisition could be done consecutively on the same cells, with a 15 min pause between the two pulse applications for resealing of the membrane and restoring low calcium concentration. We can observe a good agreement between fast and slow recording (Figure 1A), and no extra peak was seen with our fast-acquisition mode. However, we cannot rule out shorter peaks that may be seen with even faster image acquisition.

Exposing cells to single pulses of 100 ns, 40 kV/cm (Figure 1B), 1 μs, 2500 V/cm (Figure 1C), 10 μs, 1000 V/cm (Figure 1D), and 100 μs, 400 V/cm (Figure 1E) lead to comparable results. Relative Ca^2+^ concentration is increased in all cells. Relative Ca^2+^ concentration in cells gradually rises through the first few seconds after the pulse and reaches its plateau. However, the relative Ca^2+^ concentration in cells exposed to 100 ns does not reach the plateau during 10 s of image acquisition but continues to rise with a slower pace. Relative Ca^2+^ concentration in cells exposed to 1 μs, 2500 V/cm pulses should be higher with a higher electric field applied, however, the applied electric field was maximal with our exposure setup. Relative intracellular Ca^2+^ concentration increases with increasing pulse amplitude (see Section 2.4).

### 2.2. The Direction of Calcium Ions Uptake after Exposure to Pulse with Respect to the Direction of Electric Field

**Figure 2:**
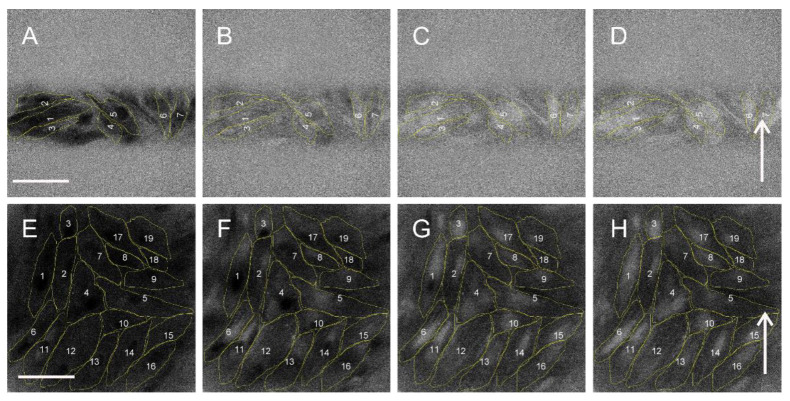
H9c2 cells exposed to a single 100 ns, 40 kV/cm (**A**–**D**) or 100 μs, 400 V/cm (**E**–**H**) electric pulse at different times with respect to pulse application: before pulse application (**A**,**E**), 1.75 s (**B**,**F**), 5 s (**C**,**G**), and 8 s (**D**,**H**) after pulse application. Figures are Fura-2 ratio 340/380 images, higher relative Ca^2+^ concentration in cells is seen as brighter. In images (**A**–**D**), cells are seen only at the 100 µm gap between gold electrodes. Scalebar: 100 µm. Arrow: the direction of the electric field.

**Figure 3:**
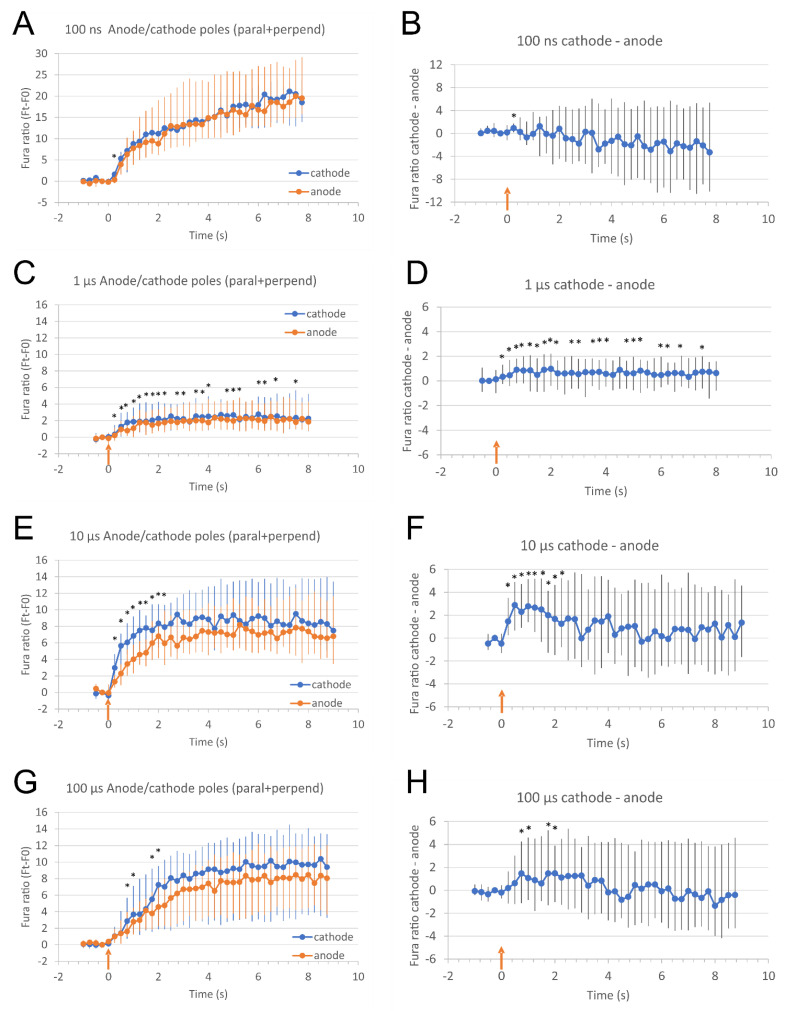
Relative calcium ion (Ca^2+^) concentration at the poles facing anode (+) and cathode (−) in cells (both parallel and perpendicular cells together) (**A**,**C**,**E**,**G**) and fura ratio difference between cathode and anode (**B**,**D**,**F**,**H**) after electroporation with a single 100 ns, 40 kV/cm (**A**,**B**), 1 μs, 2500 V/cm (**C**,**D**), 10 μs, 1000 V/cm (**E**,**F**), and 100 μs, 400 V/cm pulse (**G**,**H**) as expressed in Fura-2 ratio 340/380 over time. Relative Ca^2+^ concentration in cells was monitored with a fluorescent calcium indicator Fura-2, image acquisition was done every 250 ms. Results are presented as median from cells (only parallel and perpendicular cells) pooled from nine (**A**), six (**B**), five (**C**), and four (**D**) experiments (19–60 cells analyzed). Time of pulse application is noted with a red arrow. Vertical bars represent Q1 and Q3. *—results at cathode and anode statistically different (*p* < 0.05, Wilcoxon signed rank test).

**Figure 4:**
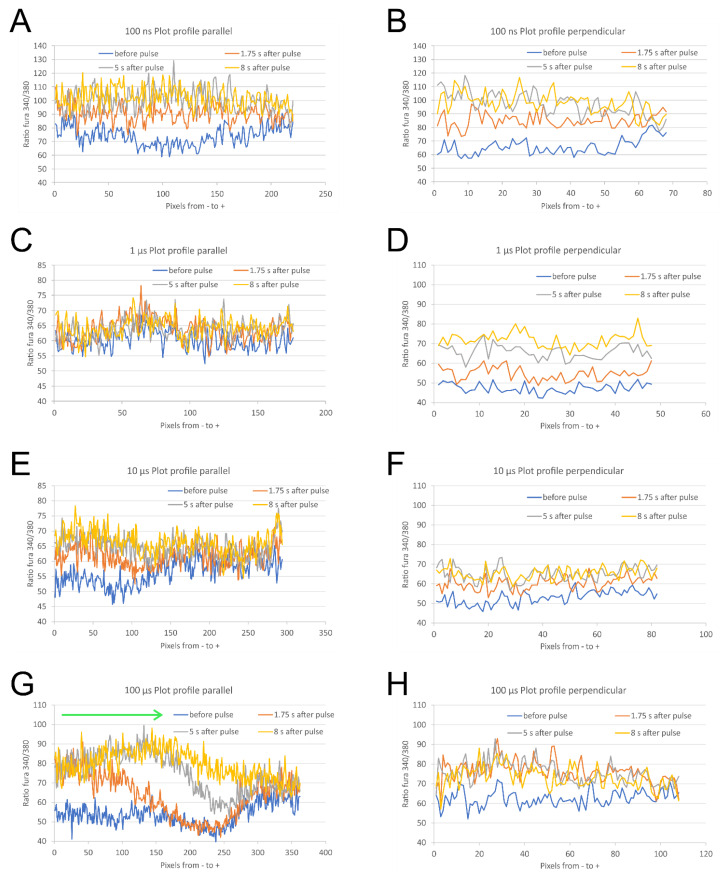
A plot profile of the cell along the longest axis parallel to electric field (from cathode (−) to anode (+) side) of relative Ca^2+^ concentration as expressed in Fura-2 ratio 340/380 of a representative parallel and perpendicular cell, at different times with respect to pulse application. Cells were exposed to a single 100 ns, 40 kV/cm (**A**,**B**), 1 μs, 2500 V/cm (**C**,**D**), 10 μs, 1000 V/cm (**E**,**F**), and 100 μs, 400 V/cm (**G**,**H**) pulse. Green arrow in (**G**) indicates the progression of Ca^2+^ ions in time.

Microscopic images revealed that after 100 ns, 40 kV/cm pulse exposure no difference in Ca^2+^ uptake between poles facing anode (+) and cathode (−) (Figure 2A–D) occured. We analyzed relative Ca^2+^ concentration in poles facing cathode (−) and anode (+) in parallel and perpendicular cells, where these regions can be easily defined. Relative Ca^2+^ concentration (expressed in Fura 340/380 ratio) in anodic (+) and cathodic (−) poles of parallel and perpendicular cells together were not significantly different, except immediately after pulse (at 0.25 s) (Figure 3A). The difference between the cathode (−) and anode (+) is even negative from 0.72 s after pulse exposure on (Figure 3B) which points to slightly higher uptake at the anode side compared to cathode, however, the differences are not significant. Plot profiles of relative Ca^2+^ concentration (expressed in Fura 340/380 ratio) after 100 ns, 40 kV/cm thus shows no apparent calcium progression in the direction of the electric field neither in parallel nor in perpendicular cells (Figure 4A,B). Experiments with 100 ns pulses were performed at room temperature and without CO_2_ incubation (in contrast with experiments with microsecond pulses that were done at 37 °C and CO_2_ incubation). Therefore, effects and contributions of different conditions on results are possible.

After exposing cells to 1 μs, 2500 V/cm pulses, the relative Ca^2+^ uptake was slightly higher at the pole facing cathode (Figure 3C,D). Due to an overall lower relative increase in Ca^2+^ concentration, the difference between cathode (−) and anode (+) is very small, yet significant.

When cells were exposed to 10 μs, 1000 V/cm (Figure 3E,F) or 100 μs, 400 V/cm pulses (Figure 2E–H and Figure 3G,H) Ca^2+^ uptake was significantly greater at the pole of the cell facing cathode (−) than at the anode (+). In cells of both orientations (parallel and perpendicular), during the first few seconds after 10 and 100 μs pulse application, relative Ca^2+^ concentration increased more at the pole facing cathode than at the anode. In cells exposed to a 10 μs pulse, Ca^2+^ concentration (expressed in Fura 340/380 ratio) was significantly higher at the pole facing cathode than at the pole facing anode from 0.25–2.25 s after pulse (0.5 s after pulse the difference between cathode and anode reached a median of 2.881, with Q1 and Q3 0.654 and 4.894, respectively, Wilcoxon signed rank test, *p* < 0.05). During the following seconds, the difference in uptake at the cell poles diminishes, Ca^2+^ diffuses throughout the cell and relative Ca^2+^ concentration at poles becomes of similar value (5.25 s after pulse application the difference between cathode and anode (Fura ratio (cathode)—Fura ratio (anode)) reached a median of −0.322, with Q1 and Q3 −2.907 and 4.808, respectively). 

In cells pulsed with 100 μs pulse, relative Ca^2+^ concentration was significantly higher 0.75–1 s and 1.75–2 s after the pulse at the pole close to the cathode than at the pole close to the anode (0.75 s after pulse the difference between cathode and anode reached a median of 1.476, with Q1 and Q3 −1.117 and 4.248, respectively, Wilcoxon signed rank test, *p* < 0.05). 

The most prominent uptake from the cathode side of the cell can be best seen on the plot profile of relative Ca^2+^ concentration (expressed in Fura ratio) from a representative parallel cell after 100 μs pulse exposure (Figure 4G): a calcium progression by diffusion in the direction from the cathode (−) side towards the anode (+) side can be clearly observed in cells oriented parallel to the electric field 1.75 s, 5 s, and 8 s after the pulse exposure (indicated by an arrow). In perpendicular cells, the calcium progression is not so evident (Figure 4H) since the distance between cathode (−) and anode (+) side of H9c2 cells is roughly 3.5 times shorter than in parallel cells [32]. The differences in relative Ca^2+^ concentration at poles after 100 μs pulse exposure are indeed more pronounced in parallel than perpendicular cells (Supplement, Figure S2G,H). Results presented in supplemental figures are also in agreement with our previous study [32]—that cells oriented perpendicular are more affected than parallel after 100 ns and 1 μs pulse exposure (Supplement, Figure S2A–D), whereas after exposing to 10 and 100 μs, cells oriented parallel to electric field are more affected than perpendicular (Supplement, Figure S2E–H). Interestingly, with 100 ns pulses, in parallel cells, Ca^2+^ uptake is slightly higher at the anode pole, and in perpendicular cells, Ca^2+^ uptake is higher at the cathode pole; however, the differences are not statistically different due to high variability of results.

### 2.3. The Source of Ca^2+^ for Calcium Concentration Elevation after Electroporation

**Figure 5:**
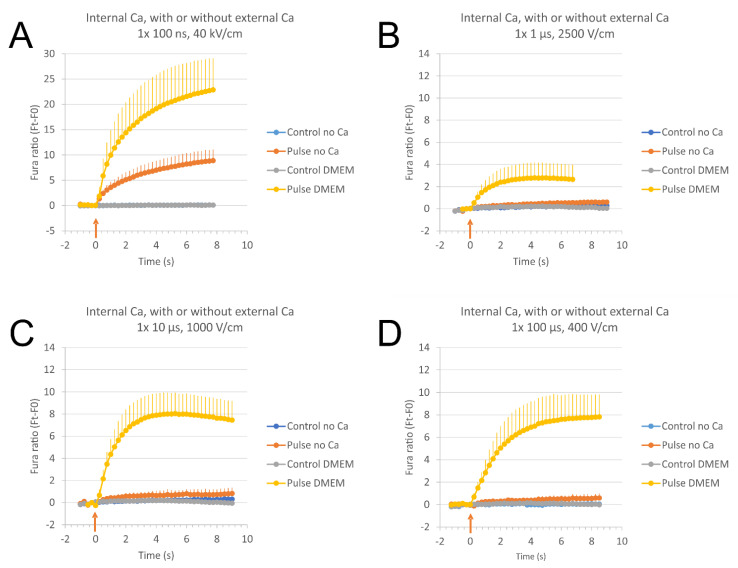
The source of calcium ions for calcium concentration elevation after electroporation. Relative calcium concentration in H9c2 cells was monitored with a fluorescent calcium indicator Fura-2 and expressed as Fura-2 ratio 340/380 over time (subtracted baseline). Image acquisition was done every 250 ms. (**A**): H9c2 cells were exposed to a single pulse of 100 ns, 40 kV/cm in conditions either without external Ca^2+^, but with EGTA present (no Ca, red) or in DMEM culture medium with Ca^2+^ present (DMEM, yellow). Results are presented as mean from six (no Ca^2+^) or nine (DMEM) experiments (5–13 cells per experiment analyzed). (**B**–**D**): The same cells were exposed to a single pulse of 1 μs, 2500 V/cm (**B**), 10 μs, 1000 V/cm (**C**), and 100 μs, 400 V/cm (**D**), first in conditions without external Ca^2+^ but with EGTA present, and after that, the medium was changed to DMEM culture medium with Ca^2+^ present. Results are presented as mean from four (**B**), five (**C**), and three (**D**) experiments (11–30 cells per experiment analyzed). Time of pulse application is noted with a red arrow. Vertical bars represent SD. Controls are experiments without pulse application.

With the use of SMEM medium without Ca^2+^, but with calcium chelator EGTA as a poration medium (conditions without external Ca^2+^), the source of Ca^2+^ ions relative Ca^2+^ concentration increase in cells after electroporation was determined (Figure 5). When cells were exposed to a single 100 ns, 40 kV/cm pulse (Figure 5A) in conditions without external Ca^2+^, the relative concentration of Ca^2+^ ions (expressed as Fura-2 ratio 340/380) in cells increased, although not to the same extent as in conditions with external Ca^2+^ (in DMEM culture medium). This means that the release of Ca^2+^ from internal stores (ER, mitochondria) occurred due to 100 ns, 40 kV/cm pulse application. In conditions with external Ca^2+^, we can however observe, in addition to Ca^2+^ released from internal stores, an uptake of external Ca^2+^ from extracellular medium. 

On the contrary, when cells were exposed to a single pulse of 1 μs (Figure 5B), 10 μs (Figure 5C), or 100 μs (Figure 5D) in SMEM medium, we did not see that Ca^2+^ increase, i.e., Ca^2+^ is not released from internal stores (no Fura response in conditions without external Ca^2+^), suggesting all of the Ca^2+^ ions that contributed to the increased relative Ca^2+^ concentration in DMEM culture medium (with Ca^2+^) are taken up from the poration medium, i.e., from the extracellular medium.

**Figure 6:**
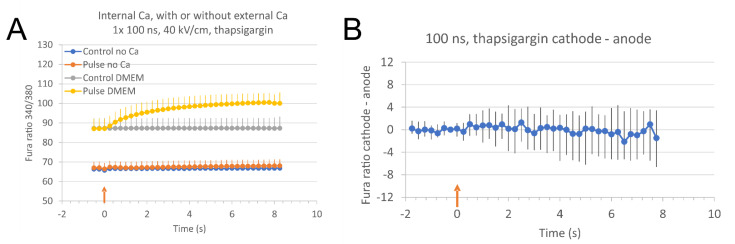
The exposure of cells incubated with 100 nM thapsigargin (for 30 min) to a single 100 ns, 40 kV/cm pulse in media with (DMEM) or without Ca^2+^ ions (SMEM and EGTA). Relative calcium concentration in H9c2 cells was monitored with a fluorescent calcium indicator Fura-2 and expressed as Fura-2 ratio 340/380 over time. Image acquisition was done every 250 ms. (**A**): Relative calcium concentration in all cells exposed to a single 100 ns pulse in different media. Results are presented as mean ± SD from ten (no Ca^2+^) or 14 (DMEM) experiments (3–11 cells per experiment analyzed). Controls are experiments without pulse application. (**B**): Fura ratio difference between cathode and anode in thapsigargin treated cells (both parallel and perpendicular cells together) after electroporation with a single 100 ns, 40 kV/cm in medium with Ca^2+^ ions (DMEM). Results are presented as median from cells (only parallel and perpendicular cells) pooled from 14 experiments (12–30 cells analyzed), vertical bars represent Q1 and Q3. Time of pulse application is noted with a red arrow.

Next, we tested whether exposure to 100 ns pulses can lead to detection of asymmetric uptake of Ca^2+^ ions if the release from internal stores is eliminated. For this purpose, we incubated H9c2 cells with thapsigargin, a well-known blocker of sarco-endoplasmic reticulum Ca^2+^-ATPases (SERCA) that passively depletes the ER [58,59]. When cells incubated with 100 nM thapsigargin were exposed to a single 100 ns pulse in a medium without calcium (SMEM with EGTA), an increase of relative internal Ca^2+^ concentration was not observed (Figure 6A). This points to a successful depletion of internal Ca^2+^ stores.

On the other hand, cells treated with 100 nM thapsigargin that were later incubated in DMEM (containing Ca^2+^ ions) showed high basal internal Ca^2+^ concentration (baseline not subtracted) that cannot be restored in these conditions for 30 min (Figure 6A). The reason for this might be due to triggering Ca^2+^ entry from the outside medium through Ca^2+^ channels of different pathways of store-operated (capacitive) Ca^2+^ entry [60]. The exposure of thapsigargin-treated cells in DMEM led to an increase of internal Ca^2+^ concentration (Figure 6A) which points to an uptake of Ca^2+^ ions from outside without a release from internal stores, however, the uptake through the channels (store-operated, other) cannot be neglected. 

We analyzed relative Ca^2+^ concentration in at the poles facing anode (+) and cathode (−) in thapsigargin treated cells (both parallel and perpendicular cells together) after electroporation with a single 100 ns, 40 kV/cm in medium with Ca^2+^ ions (DMEM) (Figure 6B). The relative Ca^2+^ concentration differences between cathode (−) and anode (+) were still not significantly different, and the values are around zero. However, for final confirming asymmetrical Ca^2+^ uptake after 100 ns pulse exposure, plasma membrane channel inhibitors should be used. 

When results were analyzed separately for parallel and perpendicular cells (Supplement, Figure S3A,B), cells parallel to electric field exhibited a symmetrical uptake, whereas cells perpendicular to electric field exhibited a slightly higher uptake at the cathode side immediately after pulsing (cathode and anode significantly different at 0.75 s after pulse exposure, Wilcoxon signed rank test, *p* < 0.05).

### 2.4. The Direction of Calcium Ions Uptake after Pulse Exposure to Low Electric Field and Reversed Polarity 100 μs Pulses

In a theoretical paper from Saulis, electroporation with pulses with low amplitude (just above the threshold for electroporation) could lead to predominantly anodal (+) EP [54]. We thus tested if we could reverse the polar asymmetry by lowering the electric field strength of 100 µs pulse to 200 V/cm.

**Figure 7:**
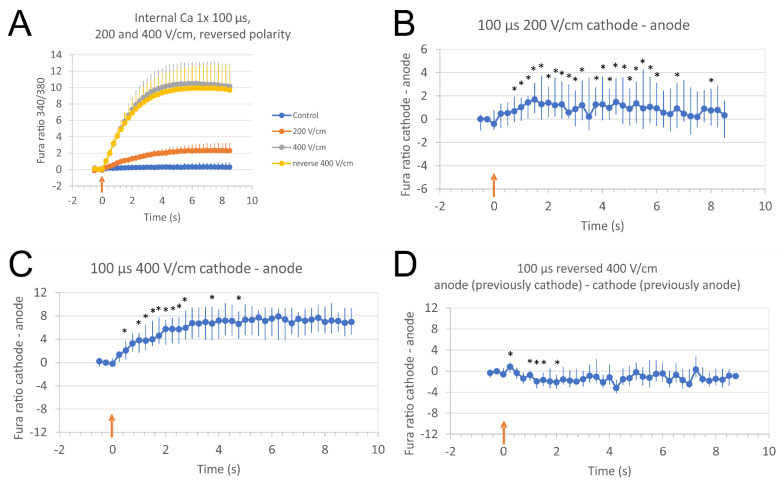
Relative Ca^2+^ concentration in cells after single, 100 µs of low electric field strength, or reversed polarity pulses. Relative Ca^2+^ concentration in cells was monitored with a fluorescent calcium indicator Fura-2, image acquisition was done every 250 ms. Time of pulse application is noted with a red arrow. (**A**): Single pulses of 100 µs were applied consecutively on the same cells (with a pause for recovery in-between): 200 V/cm (low electric field strength, red), 400 V/cm (regular, as in other experiments using 100 µs, grey), and 400 V/cm with reversed polarity (yellow). Results are presented as mean ± SD from four experiments (15–19 cells analyzed, all cells are taken into account). (**B**–**D**): Fura ratio difference between cathode and anode in cells (both parallel and perpendicular cells together) after low electric field strength (200 V/cm) (**B**), regular (400 V/cm) (**C**), or reversed polarity 400 V/cm (**D**) single pulses. Single pulses of 100 µs were applied consecutively on the same cells (with a pause for recovery in-between). In experiment D, cathode (−) from experiment C became anode (+), and anode (+) from experiment C became cathode (−). Results are presented as median from cells (only parallel and perpendicular cells) pooled from four experiments (29 cells analyzed), vertical bars represent Q1 and Q3. * Results at cathode and anode are statistically different (*p* < 0.05, Wilcoxon signed rank test).

When cells were exposed to 100 μs pulses of low electric field strength (200 V/cm), we did not observe any change of the direction of Ca^2+^ uptake (Figure 7A,B). Ca^2+^ uptake was still slightly but significantly more prominent on the cathode (−) side of cells leading to positive values of difference between cathode and anode (Fura ratio (cathode)—Fura ratio (anode)). When cells were pulsed with a regular, 400 V/cm pulse of reversed polarity, the direction of Ca^2+^ uptake was reversed (see Figure 7C,D), the uptake was still higher at cathode (anode in C) than anode (cathode in C), leading to negative values od difference between anode (cathode in C) and cathode (anode in C) (Figure 7D).

## 3. Discussion

It was described previously that orientation plays a significant role in electroporation extent when cells are exposed to electric pulses of different durations in the range from 100 ns to 100 μs [32]. We therefore further explored the effect of pulse duration on the polar uptake using these pulses.

The results of our study show that the uptake of Ca^2+^ after exposing cells to a single 1–100 µs pulse is polar and asymmetrical: it is higher at the pole facing the cathode (−) than at the pole facing the anode (+). 100 ns pulse, on the other hand, causes symmetrical Ca^2+^ uptake, with no apparent preference of poles facing to either electrode, except at first time point (250 ms after the pulse). Interestingly, Gabriel and Teissié [45] reported symmetry/asymmetry as being dependent on pulse duration, however, at a much longer range of pulse duration as in our study (at 240 µs pulse duration, they reported symmetrical uptake, whereas we have asymmetry already at 100 µs pulse). Why this difference in polar uptake (symmetry, asymmetry) exists is still not understood. It is assumed that the duration of the pulse determines the density of structures that support the transport [16,45]. It was also suggested that different resealing kinetics (slower on one side) is at least in part responsible for observed asymmetry [56].

Several other studies reported asymmetric transport at the poles of cells facing the electrodes after micro and millisecond pulse exposure. However, the preferences of the side of the cell reported in studies are different: the uptake of molecules was preferentially on the negative (cathodal) side, which is in agreement with our results [35,36,37,38,39,40,56,61], or the positive (anodal) side of cells, which is in contrast to our results [12,22,40,42,43,44,45,46,47,48,49,50,51,55] (see Table 1). 

The anodal (+) preference was explained as a consequence of superposition of induced transmembrane voltage (ITV) and resting potential: mostly, resting potential of the cells is negative inside and it adds to the transmembrane potential at the anode (+) and subtracts transmembrane potential at the cathode (−) side [12,43,47]. There are some experimental data that support but also that contradict this theory: the changes in transmembrane potential at poles were less asymmetric in Na^+^-free medium where the resting potential was lower [35], however, the asymmetry of cell injury and transport of molecules also persisted after medium change [35,51,62] or after depolarization [62], which all affects resting potential. 

The cathodal (−) preference was also explained with the superposition of ITV and resting potential. Poration that starts on the anodal side of the cell produces current flowing through the porated area, which reduces the transmembrane potential on the anode (+) side while increasing the induced transmembrane potential on the cathodal (−) side. Permeabilization on the cathode (−) side exceeds that on the anode (+) side [35,36,39,54,56], with slower resealing of membrane on cathodal (−) side [56] leading to preferentially cathodal (−) transport of molecules. Therefore, the time of transport detection is also important [39,45].

According to theoretical predictions by Saulis [54], both states of preferential uptake are possible. At a sufficiently strong electric field, the cathodic pores are larger than the anodic pores, whereas the fraction of membrane area occupied by the pores is greater in the anodic side (a small number of larger pores on the cathode-facing side and a large number of small pores at the anode-facing side). This would explain why the smaller probes (such as Ca^2+^ and ethidium bromide) appear to enter predominately through the anodic side, and the larger probes (such as ethidium homodimer and propidium iodide) enter through the cathodic side [40]. However, the theoretical analyses presented in this paper by Saulis cannot explain the origin of all the manifestations of asymmetrical electroporation that have been observed [54]. 

Saulis also predicted that if the pulse length is short enough, the pores on the cathodic (−) side do not have time to appear and can lead to predominantly anodal (+) EP, particularly with electric pulses just above the threshold for electroporation [54]. We thus tested if we could reverse the polar asymmetry with lowering the amplitude of 100 µs single pulse (200 V/cm), but failed to achieve predominantly anodic (+) Ca^2+^ uptake and thus failed to confirm his predictions.

Asymmetry was also shown to depend on the probe charge that is affected by electrochemical potential [39,55], especially in the case of larger charged molecules such as DNA that can be dragged by electrophoresis during pulses [47,61]. Ions such as Ca^2+^ can also be dragged in the direction towards the negative electrode by electrophoresis during longer pulses [22,63]. Electrophoresis cannot be solely responsible for the asymmetric transport, since the asymmetry also persists after the pulse when the electric field is switched off [45,51]. It is hypothesized that post-electroporation transport still includes a significant drift (electrophoretic) component after the application of electric pulses [64]. Electrophoresis is also not the case with our results, since Ca^2+^ drag in the direction towards the negative electrode would lead to preferentially anodal (+) uptake. The unidirectional flow through electropores could also be caused by electroosmosis during the pulse, when ionic aqueous medium causes a net hydrodynamic flow toward the electrode with the polarity opposite to the dominant charge in the medium [37,62].

Asymmetric polar transport may depend on asymmetric membrane structures that can behave in a different way on different sides [35,39,45], and may vary among different cell types [35]. In the present study, we used H9c2 rat cardiac myoblast cell line. Studies in which authors investigated polar asymmetric transport in cardiac cells exposed to electric pulses of different durations have all used isolated, primary cardiomyocytes and they all reported preferentially anodal (+) uptake of molecules [22,46,53,65], which is contrary to our results. H9c2 cell line shows many morphological and physiological characteristics typical for cardiac cells [66,67,68], however their differences may account for different results in EP of primary cardiomyocytes and cardiac cell lines [32]. However, different cell types cannot be solely responsible for observed different molecular transport, since the EP in the same conditions led to similar results in different cell types (CHO, HeLa, and NIH 3T3) [40].

Poration medium composition was also suggested to affect polar asymmetry in transport of molecules. Tekle et al. observed a reversal of polar preference of ethidium bromide and Ca^2+^ transport when the poration medium was changed from low salt medium to high salt medium. This reversal was explained by rapid resealing of membrane pores in high ionic strength medium. Consequently, the smaller sized pores at the anode-facing side would close rapidly, and thus the probes can enter only through the cathodal (−) pole [40]. Djuzenova et al. also reported that medium composition affects polar asymmetry, via changing cell resting potential, and possibly by changing the mechanical properties of membrane (surface tension, viscous drag, inertia, etc.), and thus affecting widening of pores in the plasma membrane [43]. Our experiments were done in culture medium, which is considered a high salt medium (as opposed to low conductivity media used frequently in EP experiments), and results are consistent with [40] that reported preferentially cathodal (−) uptake of Ca^2+^ in high salt medium. On the other hand, Semenov et al. exposed embryonic rat cardiac myocytes to a single 4 ms pulse in a physiological saline that was also high salt medium, and reported preferentially anodal (+) uptake of Ca^2+^ [22]. The reason for this discrepancy remains obscured.

When H9c2 cells were exposed to a single 100 ns pulse, an asymmetry of polar transport was not observed, except at the first time point 250 ms after the pulse. In other studies, similar to those that investigated EP with µs and ms pulses, all possible outcomes were reported: symmetrical [22,39,49], preferentially anodal (+) [39,41,48,52,53], and cathodal (−) [26] (see Table 1). Since the experiments with 100 ns pulses were done at room temperature and without CO_2_ incubation (in the case of microsecond pulses, the experiments were done at 37 °C and CO_2_ incubation), this may have affected our results. 

Preferentially anodal asymmetry with ns pulses was explained again with superposition of ITV and resting potential, the differences in pore density and size on poles, cylindrical nanopores with nonuniform cross section, and also by field-driven translocation of phospholipids [69]. Anionic phospholipids such as phosphatidylserine (PS) are electrostatically driven across the bilayer boundary at the periphery of a pore in the direction of the anode, which results in PS “flopping” (externalization) at the anode (+) and “flipping” (internalization) at the cathode (−) side, which accounts for preferentially anodic (+) externalization of PS [49,51,52,70]. This can affect membrane bilayer asymmetry and domain organization, as well as membrane-solute interactions (such as PS attraction of PI and YO-PRO-1), and thus may have an impact on long-term transport after pulse exposure, leading to a preferentially anodic (+) uptake of molecules after exposure of cells to ns pulses [39]. We, however, did not detect preferentially anodal (+) uptake of Ca^2+^. nsEP caused more pronounced asymmetry in uptake of Ca^2+^ than Ba^2+^, which may be caused by local amplification of Ca^2+^ by store disruption, CICR, store-operated Ca^2+^ entry, or some other process or attenuation at the opposite pole by sequestration of Ca^2+^ into the ER [41].

As we can see in Figure 5A, Ca^2+^ is released from internal stores, as seen by an increase of relative internal Ca^2+^ concentration after 100 ns pulse exposure to cells in the medium without Ca^2+^. This means that the release of Ca^2+^ from the internal stores can be the reason behind the absence of asymmetrical transport. Sun et al. also speculated that intracellular disturbances accounted for observed symmetrical Ca^2+^ uptake in spite of preferentially anodal (+) PS translocation [49]. It is known that electric pulses of nanosecond duration cause a release of Ca^2+^ from internal stores, mainly endoplasmic reticulum (ER) [26,27,28]. Myocytes have a highly regulated intracellular Ca^2+^ cycling system [53]. Symmetrical Ca^2+^ transport was also observed in cardiac cells after ns pulse exposure where internal Ca^2+^ stores were depleted [22], so at least in these experiments, the release of Ca^2+^ from internal stores was not the reason for symmetry. 

The increase of internal Ca^2+^ can be further amplified with store-operated (capacitive) Ca^2+^ entry (SOCE) or Ca-induced Ca^2+^ release (CICR) from internal stores which may also account for the absence of asymmetrical transport. It was suggested that nsEP cause a depletion of Ca^2+^ stores in ER (by electroporation of internal membranes, direct effect on protein channels and/or by mimicking a ligand signal that could trigger receptors on internal membranes), which in turn activates plasma membrane calcium influx through channels linked to SOCE [31]. However, it was later proposed that nsEP nanoporation (an occurrence of pores of nm size, too small for PI entry) of plasma membrane can account for the influx originally thought to be the capacitive calcium entry [30]. The second amplifying mechanism, CICR, was shown to activate above a certain nsEP amplitude threshold. The electroporation of plasma membrane and/or ER caused a substantial increase in internal Ca^2+^ concentration, which led to inositol trisphosphate receptor (IP_3_R) -dependent release from ER [29,30]. These complex mechanisms of calcium regulation could have a profound effect on spatial distribution of calcium ions in a cell after nsEP. However, with the use of a cocktail of verapamil (for blocking L-type Ca^2+^ channels in plasma membrane), caffeine (for depleting Ca^2+^ from the ER by stimulating ryanodine receptors), and cyclopiazonic acid (for blocking re-uptake of Ca^2+^ to the ER) in the paper from Semenov [22], CICR is unlikely to be responsible for symmetrical transport after nsEP. In our experiments, we also cannot rule out the possibility that nsEP affect permeability of protein ion channels directly [22,23,71], and that this may be done more uniformly across the cell, however, the direction of electric field may influence the effect of electric field on ion channels [71].

Nevertheless, no one observed (at least not to our knowledge) the polar uptake of Ca^2+^ after nsEP in both normal cells and cells with depleted internal stores of Ca^2+^. We performed these experiments with the use of thapsigargin, a known inhibitor of SERCA pumps that enables a depletion of internal Ca^2+^ stores [58,59]. In thapsigargin-treated cells, however, the uptake was still symmetrical. Therefore, the release of Ca^2+^ from the internal stores (ER) may interfere with asymmetrical Ca^2+^ uptake after 100 ns pulse exposure but does not solely account for a symmetrical increase of Ca^2+^ throughout the whole cell. The thapsigargin treatment also triggers additional Ca^2+^ uptake from outside medium through store-dependent and store-independent channels in plasma membrane contributing to different pathways of store-operated Ca^2+^ entry (SOCE) [60], which also interferes with asymmetrical transport of Ca^2+^ through permeabilized membrane. This was also the case in our experiments, since the base level of internal Ca^2+^ remained high (Figure 6A). Therefore, for final confirmation of asymmetrical Ca^2+^ uptake after 100 ns pulse exposure, plasma membrane channel inhibitors should be used.

The uptake of Ca^2+^ ions after EP is important due to their signaling role, regulating many cellular functions [19]. Moreover, Ca^2+^ can function as a stressor in cell damage, death, and survival [72]. Calcium is also important for triggering cell membrane repair [73,74]. Ca^2+^ acts locally [19,72], therefore it is of major importance to determine the spatial distribution of high Ca^2+^ concentration in cells after electroporation. This can be very important in tissues with elongated cells since it was shown that orientation of these cells in electric field affects extent of electroporation [32]. A different extent of electroporation at different parts of elongated cells such as muscle or cardiac cells may have an impact on electroporation-based treatments such as drug delivery, pulse-field ablation, and gene electrotransfection, but the impact is yet to be determined.

To summarize, we confirmed that pulse duration affects polar uptake of Ca^2+^ into cells, however, in the case of 100 ns pulses, Ca^2+^ release from internal stores (ER) or other more complex pathways of Ca^2+^ control (e.g., SOCE) may play an important role in asymmetrical transport detection. Moreover, we did not confirm the hypothesis that, with the use of low amplitude 100 µs pulses, we can achieve the reversal of polar asymmetric Ca^2+^ uptake [54].

## 4. Materials and Methods

### 4.1. Cells

H9c2 rat cardiac myoblast cell line (European Collection of Authenticated Cell Cultures ECACC 88092904) was used in experiments. They were selected because we already studied the effect of orientation on electroporation on this cell line [32], and are of elongated shape. Moreover, this study is important for optimizing treatments of heart arrhythmias by ablation with electroporation [3]. For nanosecond pulse exposures, H9c2 cells were seeded in culture medium Dulbecco’s Modified Eagle Medium DMEM D6546 (Sigma-Aldrich, Darmstadt, Germany) supplemented with 4 mM L-glutamine (Sigma), 10% FBS (Sigma) and antibiotics penicillin 1 U/mL (PAA, Toronto, Canada), streptomycin 1 µg/mL (PAA) and gentamycin 50 µg/mL (Sigma) in a humidified chamber at 37 °C and 10% CO_2_ atmosphere on a 12 mm diameter coverglass in 24-well plates one or two days before the experiment at 5 × 10^4^ and 3 × 10^4^ cells per well, respectively. For microsecond pulse exposures, H9c2 cells were seeded in their culture medium in two well Lab-Tek chambered coverglass (Nunc, ThermoFisher Scientific, Waltham, MA, USA) one or two days before the experiments at 8 × 10^4^ and 5 × 10^4^ cells per well, respectively.

### 4.2. Detection of Cell Relative Calcium Concentration with Fura-2 AM

Relative intracellular calcium concentration was detected using the fluorescent calcium indicator Fura-2 acetoxymethyl (AM) ester (ThermoFisher Scientific, Waltham, MA, USA). When calcium concentration is elevated, the Fura-2 dye changes the fluorescence spectrum, which can be detected with ratiometric measurements [18,21].

H9c2 cells were stained with 2 µM Fura-2 AM in their culture media (DMEM with supplements, see Section 4.1 Cells) at 37 °C for 30 min. Fura-2 AM is a cell-permeant form of the dye that enters the cell and is then cleaved by cell esterases into a cell-impermeant form. After staining, cells were washed three times with fresh DMEM. Electroporation was performed in fresh DMEM culture medium. Cells were monitored under an inverted epifluorescent microscope (Zeiss Axiovert 200, Zeiss, Oberkochen, Germany) using 40× objective (LD Achroplan 40×/0.60 corr, Zeiss) and Prime sCMOS camera (Photometrics, Tucson, AZ, USA). For microsecond pulse exposures, the microscope stage was heated (37 °C), and a humidified CO_2_ chamber was used to enable consecutive pulse application to the same cells during a long period of time (up to 30 min) under controlled conditions. For nanosecond pulse exposure, the microscope stage heating and CO_2_ chamber were not used in order to prevent the drying of the sample (cells on coverglass on top of microelectrodes). Cells were illuminated with a monochromator (VisiChrome Polychromator, Visitron, Puchheim, Germany) with Xe light at two excitation wavelengths (340 nm and 380 nm) and an exposure time of 100 ms for both wavelengths, using appropriate filter set (Chroma 71500: 400 dclp BS 45° D510/40 m EM 0°, Chroma Technology Corporation, Bellows Falls, VT, USA), therefore, the emission wavelength was detected at 510 nm. Images were acquired with the software VisiView (Visitron) in a time-lapse acquisition mode (fast-acquisition: total duration of image acquisition: 10 s, time interval between images: 250 ms, number of recorded images: 41). Pulses were delivered during the first two seconds of image acquisition and were later adjusted to the same pulse delivery time (see Image analysis). The results from 100 µs pulse application were compared to slow-acquisition mode (exposure time of 100 ms, total duration of image acquisition: 70 s, time interval between images: 5 s, number of recorded images: 15) as described previously [32]. Slow-acquisition mode was done on the same cells 15 min after fast-acquisition, allowing the cells to recover. The configuration for nanosecond pulses did not allow consecutive pulse application to the same cells due to the drying of the sample, therefore, slow acquisition of images was not performed.

Background (no cells) was subtracted from the images. The ratio images were obtained by the following procedure: fluorescence intensity of each pixel in images excited at 340 nm was divided by that of images excited at 380 nm. Fura-2 340/380 ratio in all pixels was multiplied by a VisiView preset factor of 100 to add numerical precision to 16-bit images with integer pixel values. Increased internal calcium concentration resulted in increased Fura-2 340/380 ratio. 

### 4.3. Exposure of Cells to Electric Pulses

Cells were exposed to electric pulses using two different electrode configurations, gold microelectrodes for delivering a single, 100 ns, 40 kV/cm pulse, and Pt/Ir wire electrodes for delivering single pulses of 1 μs, 2500 V/cm, 10 μs, 1000 V/cm, and 100 μs, 400 V/cm [21] to reach a comparable Fura-2 response (all cells are electroporated), as described previously [32]. A high electric field strength at applying 100 ns pulses (40 kV/cm) was not achievable with wire electrodes, therefore, gold microelectrodes with a narrow gap (100 µm) had to be used instead of wire electrodes (4 mm gap) (for electrodes see Supplement Figure S1A,B). When applying 100 ns pulses, anode (+) was left, when applying 1–100 μs pulses anode (+) was down, except in experiments of reversed polarity (anode (+) was up). However, when images are presented in this paper (Figure 2) we flipped the images from 100 ns EP, so anode (+) was also at the lower end of the images (down). 

For 100 ns pulses, Fura-2 labeled cells on round coverglass with cells facing down were placed on 40 µL drop of DMEM medium on top of gold microelectrodes, 2.1 µm thick, mounted onto a cover glass holder, with a gap of 100 µm between the electrodes [75]. The coverglass was gently pushed down until the distance between coverglass with cells and the electrodes reached between 30 and 50 µm, with an average distance of 40 µm, as determined by Prior ProScan III z-axis controller (Prior Scientific Ltd., Cambridge, UK). Pulses were delivered by the laboratory prototype Blumlein generator (University of Ljubljana) [76] with 1 kV RF (radiofrequency) MOSFET switch (DE 475-102N20A, IXYS-RF, USA) and two 10 m, RG 58/U, 50 Ω transmission lines (Amphenol RF). Transmission lines were directly soldered to the microelectrodes, and 270 Ω resistor (TSF 270RJE, Ohmite) was used in parallel to the load to match the generators’ and loads’ impedance. Voltage was measured on the electrode level by an oscilloscope (WaveSurfer 422, Teledyne LeCroy, Chestnut Ridge, USA) and a high-voltage probe (PPE2KV, Teledyne LeCroy). Due to the positioning of cells on top of the electrodes, the exact electric field to which the cells were exposed was determined by numerical modeling of electric field distribution around the electrodes as described previously [32], 600 V applied led to 40 kV/cm at the position of cells (Supplement Figure S1C). Briefly, electric field between the electrodes was modeled in Comsol Multiphysics v5.6 (Comsol AB, Stockholm, Sweden) using the Electric Currents physics and the stationary study. The geometry was modeled as a rectangle that was 40 µm wide and 140 µm long. The electrodes were obtained by partitioning the bottom boundary of the rectangle, with the distance between the inner edges of electrodes being set at 100 µm and the length of the electrodes being 20 µm. To the rectangle, i.e., electroporation medium, we assigned a conductivity of 1.6 S/m. Cells were exposed to the electric by putting a glass slide on the electrodes. There, the calculated electric field was approximately 40 kV/cm.

For electroporation with 1, 10, and 100 µs pulses, two parallel Pt/Ir wire electrodes, with 0.8 mm diameter and 4 mm distance between inner edges, were placed to the bottom of the Lab-Tek chamber. Pulses were delivered by a laboratory prototype pulse generator (University of Ljubljana) based on H-bridge digital amplifier with 1 kV RF MOSFETs (DE275-102N06A, IXYS, Milpitas, CA, USA) [32,50,77] for 1 µs pulses or the Electro cell B10 electroporator (BetaTech, Saint-Orens-de-Gameville, France) for 10 and 100 µs pulses. Voltage and current were measured by the oscilloscope (WaveSurfer 422) using a differential voltage probe (ADP305) and a current probe (CP030), all from LeCroy. The electric field to which the cells were exposed was approximated as a ratio of voltage to distance between the inner edges [50]. The waveforms of pulses of the same duration in the same cell exposure setups, however of slightly different electric field strengths, are shown in our previous publication [32].

To identify the source of calcium ions for elevated internal calcium concentration, we performed the following experiments. Cells dyed with Fura-2 were exposed to a single electric pulse of the same duration and electric field strength (1 μs, 2500 V/cm; 10 μs, 1000 V/cm; or 100 μs, 400 V/cm), but in calcium-depleted medium—SMEM medium—without Ca^2+^ (Minimum Essential Medium Eagle, Spinner Modification, Sigma Merck M8167) and supplemented with 5 μM calcium chelator EGTA (ethylene glycol-bis(β-aminoethyl ether)-N,N,N′,N′-tetracetic acid). After 12 min, the medium was changed to DMEM medium (contains 1.8 mM Ca^2+^), and 13–15 min after the first pulse, cells were exposed to the same pulse again. However, for 100 ns, 40 kV/cm pulses, changing the medium was not possible due to the setup configuration (cells on coverglass on top of the electrodes). Therefore, we exposed the cells to 100 ns electric pulses only in SMEM without Ca^2+^ and supplemented with EGTA and compared these results to the ones performed in DMEM medium [32]. For depleting internal Ca^2+^ stores (ER), cells were incubated with 100 nM thapsigargin during Fura-2 labeling (for 30 min in DMEM medium).

### 4.4. Image Analysis

Image analysis was done using an open-source image-processing program ImageJ (National Institutes of Health, Bethesda, MD, USA) and Excel (Microsoft, Redmond, USA). Cells in ratio images were manually encircled and mean fluorescence intensity ratio Fura-2 340/380 was determined for each cell. Time of pulsing was determined from graphs as the last time point where fluorescence is at the baseline. Since pulses were applied manually, the separate experiments were adjusted to the same pulsing time when averaged (number of experiments: 3–9) or compared to slow-acquisition mode. The baseline of each cell (fluorescence at time 250 ms before pulse exposure—the last time point before pulsing) was subtracted from each time point.

For determining the direction of calcium with respect to the position of electrodes, cells from separate experiments were pooled from the experiments and analyzed. The fluorescence intensity ratio Fura-2 340/380 was determined in manually encircled regions facing the anode (+) or the cathode (−) only in parallel and perpendicular cells since the regions can be best determined in these cells. The orientation of cells in electric field—parallel and perpendicular—in all experiments including controls was determined with ImageJ, as described previously [32]. In brief, cells that were previously encircled were fitted to an ellipse. For parallel and perpendicular cells, two conditions had to be met. First, the longer axis (a) was at least twice as long as the shorter axis (b): a > 2b. This means that the cells were substantially elongated and not round. Second, their longer axis (a) was parallel (0° ± 20° from the main axis) or perpendicular (90° ± 20° from the main axis) to the electric field. Results are presented as fluorescence in regions (with subtracted baseline) and the differences between the cathode and anode region (Fura ratio (cathode)—Fura ratio (anode)). If a difference is a positive value, the uptake is higher on the cathode side, and if a difference is a negative value, the uptake is higher on the anode side.

Plot profiles for Figure 4 were made using ImageJ along the longest axis parallel to electric field (from cathode (−) to anode (+) side) in representative cells (in experiments where both parallel and perpendicular cells were analyzed). Raw data are presented (without subtracted baseline).

### 4.5. Statistical Analysis

Statistical analysis was performed using Excel and SigmaPlot 11.0 (Systat Software, Chicago, IL, USA). The results in figures and the text are expressed as means ± SD. Significant differences (*p* < 0.05) in fluorescence intensity ratio Fura-2 340/380 between poles facing anode (+) and cathode (−) were determined by Wilcoxon signed rank test (results did not have a normal distribution).

## 5. Conclusions

The orientation of cells exposed to electric field is important, especially in elongated cells. The uptake of Ca^2+^ at cellular poles, with respect to electric field, is different, and can lead to different impacts of electroporation on cells in tissues. The molecular transport across the plasma membrane is complex, as it is affected by several different parameters and contributing factors such as pulse parameters and resting potential, leading to different pore size and dynamics, electrochemical potential, electrophoresis, electroosmosis, field-driven translocation of phospholipids, poration media, and cell types. It results in all three states regarding polar uptake: symmetrical, asymmetrical preferentially anodal (+), or cathodal (−), but the phenomenon needs further research for optimization of drug delivery techniques.

## Figures and Tables

**Table 1:** References reporting (a) symmetrical uptake of molecules. Preferential electrode uptake: C− (cathode), A+ (anode), symmetrical.

Reference	Pulse Duration Range	Pulse Parameters	Detection Molecule/Ion	Preferential Electrode Uptake	Cells
Beier 2012 [26]	ns	1 × 600 ns, 50 kV/cm	Ca^2+^	C−	NG108-15 rodent neuroblastoma
Semenov 2015 [22]	ns, ms	1 × 10 ns, 270 kV/cm	Ca^2+^	Symmetrical	Embryonic rat cardiac myocytes
		1 × 4 ms, 1100 V/cm	Ca^2+^	A+	
Bo 2020 [41]	ns	1 × 600 ns, 10 kV/cm	Ca^2+^, Ba^2+^	A+	HEK human epithelial kidney cells
Sözer 2018 [39]	ns, µs	1 × 6 ns, 200 kV/cm	YO-PRO-1, PI	A+	U-937 human histiocytic lymphoma monocyte
		1 × 6 ns, 200 kV/cm	Calcein	Symmetrical	
		1 × 220 µs, 2.5 kV/cm	YO-PRO-1, PI	C−	
		1 × 220 µs, 2.5 kV/cm	Calcein	Symmetrical	
Sun 2006 [49]	ns, ms	1 × 30 ns, 25 kV/cm	Ca^2+^	Symmetrical	Jurkat Human T lymphocytes
		1 × 5 ms, 1 kV/cm	PI	A+	
Vernier 2006 [52]	ns	100 × 4 ns, 80 kV/cm, 1 kHz	YO-PRO-1	A+	Jurkat Human T lymphocytes
Michel 2020 [48]	ns, µs	1 × 300 ns, 4.8–8.4 kV/cm 1 × 100 μs, 480–720 V/cm 8 × 100 μs, 240–480 V/cm, 5 kHz	Ca^2+^, YO-PRO-1	A+	CHO Chinese hamster ovary cells
Wang 2009 [53]	ns	4 ns, 10–80 kV/cm	Ca^2+^	A+	Rat ventricular myocytes
Djuzenova 1996 [43]	µs	Exponentially decaying pulse, time constant of 40 μs, 2–6 kV/cm	PI	A+	Sp2/0-Agl4 murine myeloma
Gabriel 1997 [45]	µs, ms	1 × 240 µs, 1.5 kV/cm	PI	Symmetrical	CHO Chinese hamster ovary cells
		1 × 0.5–3 ms, 1.5 kV/cm	PI	A+	
		1 × 20 ms, 500–1200 V/cm	PI	A+, C−	
Guionet 2018 [12]	µs	6–278 × 10 μs, 270–1800 V/cm, 1 Hz	Ca^2+^	A+	HeLa human cervical cancer
Hibino 1991 [36]	µs	1 × 10 μs, 100 V/cm	Ca^2+^, envelope	C−	Sea urchin egg
Hibino 1993 [35]	µs	1 × 100 μs, 400 V/cm	Ca^2+^, envelope	C−	Sea urchin egg
		1 × 400 μs, 400 V/cm	Ca^2+^, envelope	A+	
Kinosita 1991 [56]	µs	1 × 400 μs, 400 V/cm	Ca^2+^	C−	Sea urchin egg
Mehrle 1985 [47]	µs	1 × 10 µs, 2.3 kV/cm 1 × 20 µs, 1 kV/cm 1 × 40 µs, 1 kV/cm 1 × 60 µs, 500 V/cm	Ethidium bromide, berberine hemisulfate, fluorescein diacetate	A+	Oat mesophyll protoplasts
Sowers 1988 [37]	µs	Exponentially decaying pulse, 600 μs ms decay halftime, 7 kV/cm	FITC dextran	C−	Human erythrocyte ghosts
Tekle 1990 [55]	µs	1 × 400 μs, 5 kV/cm	Ethidium bromide	A+	NIH 3T3 fibroblasts
Tekle 1994 [40]	µs, ms	1 × 250 μs, 1.2 kV/cm (similar results also for 10 μs and 1 ms pulses)	PI, ethidium homodimer EthD-1	C−	CHO, HeLa, NIH 3T3
			Ethidium bromide, Ca^2+^ in high salt medium	C−	
			Ethidium bromide, Ca^2+^ in low salt medium	A+	
Sweeney 2016 [50]	µs	100 × 100 μs, 1250 V/cm, 2 kHz 200 × 100 μs, 750 V/cm, 2 kHz	PI	A+	CHO Chinese hamster ovary cells
Cheek 2004 [42]	ms	1 × 10 ms, 10 V/cm	PI	A+	Rat ventricular myocytes
Gabriel 1999 [44]	ms	1 × 1.2–16 ms, 1.2 kV/cm	Ca^2+^ leak out, PI	A+	CHO, HeLa
Klauke 2010 [46]	ms	1 × 4 ms, 40–68 V/cm	Ca^2+^, SNARF-1 dextran	A+	Primary rabbit ventricular myocytes
Reberšek 2007 [61]	ms	8 × 1 ms, 100–400 V, 1 Hz	DNA plasmid	C−	CHO Chinese hamster ovary cells
Sowers 1986 [38]	ms	1 × 1.2 ms, 7 kV/cm	FITC dextran	C−	Human erythrocyte ghosts
Teruel 1997 [51]	ms	1 × 5–200 ms, 167–340 V/cm	Ca^2+^	A+	2H3 rat basophilic leukemia cells, neocortical neuroblastoma cells

## Data Availability

All data generated or analyzed during this study are included in this published article (and its supplementary information files).

## Supplementary Materials

The following are available online, Figure S1: Electrodes and electric field used in the study. Figure S2: Fura ratio difference between cathode (−) and anode (+) in parallel (A,C,E,G) and perpendicular cells (B,D,F,H) after electroporation with a single 100 ns, 40 kV/cm (A,B), 1 μs, 2500 V/cm (C,D), 10 μs, 1000 V/cm (E,F), and 100 μs, 400 V/cm (G,H) pulse as expressed in Fura-2 ratio 340/380 over time (cathode–anode). Figure S3: Fura ratio difference between anode (+) and cathode (−) in parallel (A) and perpendicular thapsigargin-treated cells (B) after electroporation with a single 100 ns, 40 kV/cm pulse, as expressed in Fura-2 ratio 340/380 over time (cathode–anode).

## Sample Availability

Samples of the compounds are not available from the authors.

## Abbreviations

EP	electroporation
PI	propidium iodide
VGCC	voltage-gated calcium channels
nsEP	electroporation with nanosecond electric pulses
AM	acetoxymethyl
EGTA	ethylene glycol-bis(β-aminoethyl ether)-N,N,N′,N′-tetraacetic acid
PS	phosphatidylserine
ER	endoplasmic reticulum
ITV	induced transmembrane voltage
SOCE	store-operated (capacitive) Ca^2+^ entry
CICR	Ca-induced Ca^2+^ release
