# Supplementary material for: Pulse Duration Dependent Asymmetry in Molecular Transmembrane Transport Due to Electroporation in H9c2 Rat Cardiac Myoblast Cells In Vitro
Source: Molecules. 2021 Oct 30;26(21):6571. doi: 10.3390/molecules26216571 (PMC8588460; doi:10.3390/molecules26216571)
Supplement: Supplementary file 1 [file molecules-26-06571-s001.zip › molecules-1387640-SI.pdf]

molecules

MDPI

Article

Pulse Duration Dependent Asymmetry in Molecular Transmembrane Transport due to Electroporation in H9c2 Rat Cardiac Myoblast Cells In Vitro

Tina Batista Napotnik * and Damijan Miklavčič

Faculty of Electrical Engineering, University of Ljubljana, Tržaška cesta 25, 1000 Ljubljana, Slovenia; Damijan.Miklavcic@fe.uni-lj.si

* Correspondence: Tina.BatistaNapotnik@fe.uni-lj.si; Tel.: +386-1-4768-771

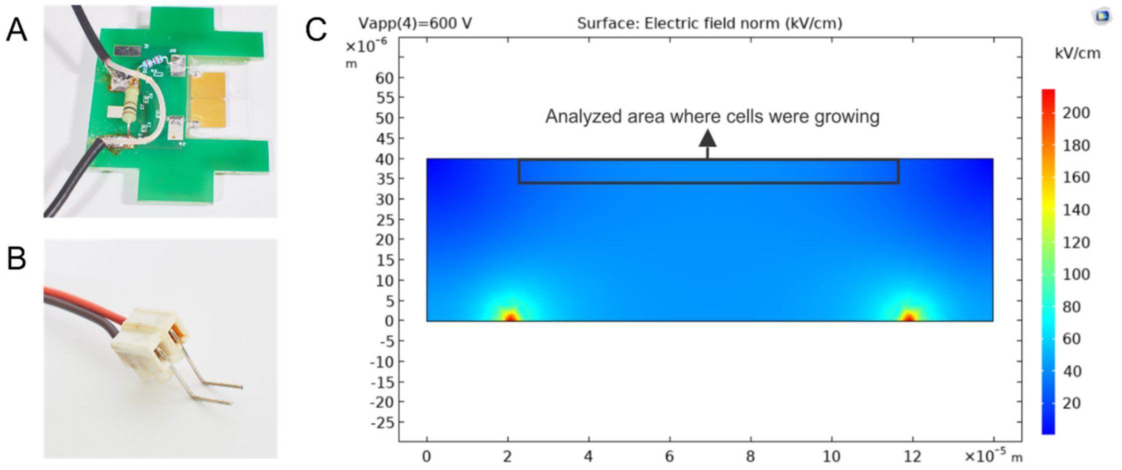

Figure S1. Electrodes and electric field used in the study. Electrodes for (**A**) 100ns pulse application, and (**B**) for 1 µs – 10ms pulse application. C: Electric field distribution in kV/cm when 600 V are applied to the electrode setup for nanosecond pulse application. Electric field between the electrodes was modeled in Comsol Multiphysics v5.6 (Comsol AB, Sweden) using the Electric Currents physics and the stationary study. Cells were exposed to the electric by putting a glass slide on the electrodes. There, the calculated electric field was approximately 40 kV/cm. Courtesy of Janja Dermol-Černe, University of Ljubljana, Faculty of Electrical Engineering.

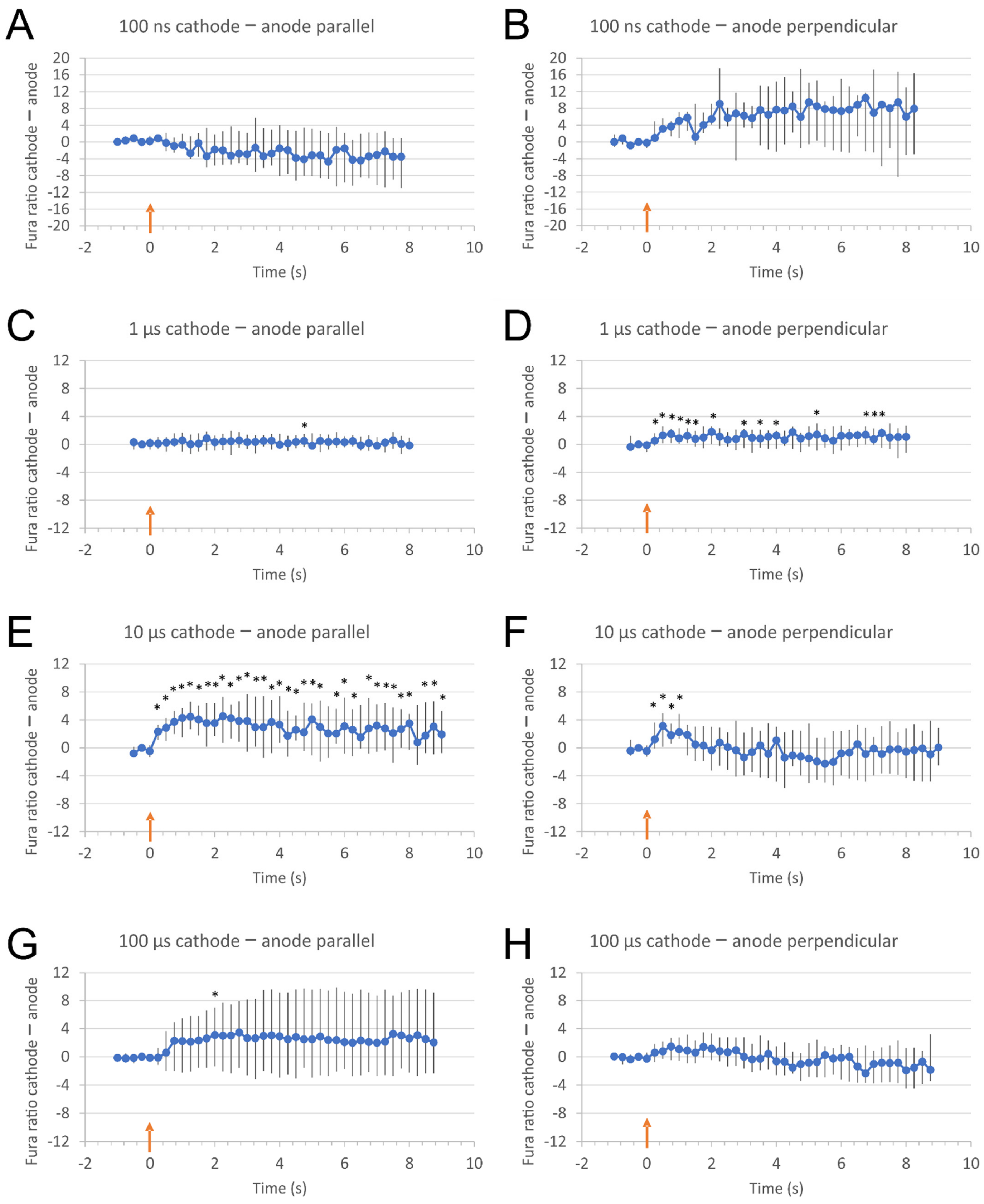

Figure S2. Fura ratio difference between cathode (−) and anode (+) in parallel (**A**, **C**, **E**, **G**) and perpendicular cells (**B**, **D**, **F**, **H**) after electroporation with a single 100 ns, 40 kV/cm (**A**, **B**), 1 µs, 2500 V/cm (**C**, **D**), 10 µs, 1000 V/cm (**E**, **F**), and 100 µs, 400 V/cm (**G**, **H**) pulse as expressed in Fura-2 ratio 340/380 over time (cathode – anode). Relative calcium concentration in cells was monitored with a fluorescent calcium indicator Fura-2, image acquisition was done every 250 ms. Time of

pulse application is noted with a red arrow. Results are presented as median from cells pooled from nine (**A**, **B**), six (**C**, **D**), five (**E**, **F**), and four (**G**, **H**) experiments (**A**: 14, **B**: 5, **C**: 32, **D**: 28, **E**: 17, **F**: 22, **G**: 14, **H**: 20 cells analyzed). Vertical bars represent Q1 and Q3. * - results at cathode and anode statistically different ($p < 0.05$, Wilcoxon signed rank test).

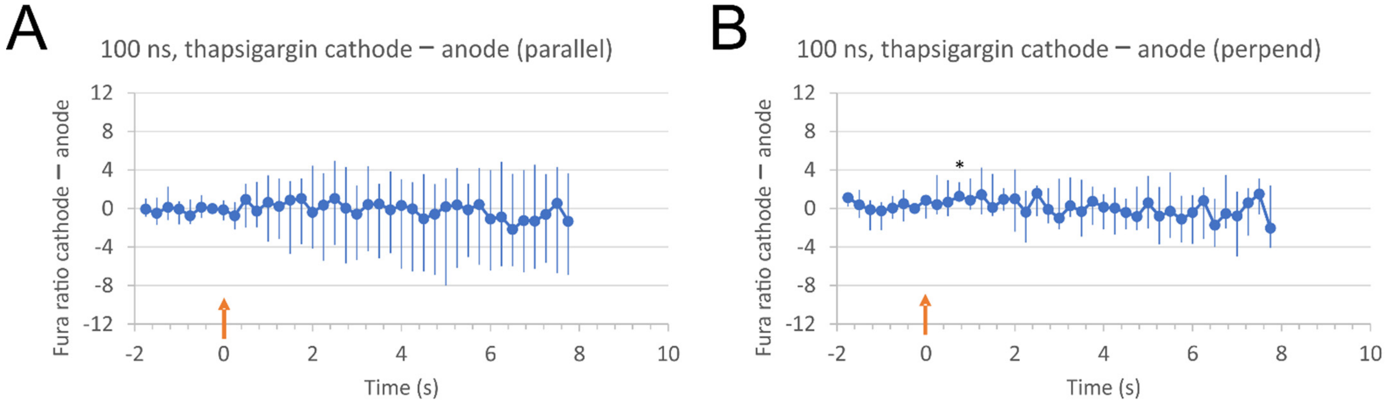

Figure S3. Fura ratio difference between anode (+) and cathode (−) in parallel (**A**) and perpendicular thapsigargin-treated cells (**B**) after electroporation with a single 100 ns, 40 kV/cm pulse, as expressed in Fura-2 ratio 340/380 over time (cathode – anode). Relative calcium concentration in cells was monitored with a fluorescent calcium indicator Fura-2, image acquisition was done every 250 ms. Time of pulse application is noted with a red arrow. Results are presented as median from cells pooled from 14 experiments (30 parallel and 12 perpendicular cells were analyzed). Vertical bars represent Q1 and Q3. * - results at cathode and anode statistically different ($p < 0.05$, Wilcoxon signed rank test).
